# Targeting chemotherapy-resistant leukemia by combining DNT cellular therapy with conventional chemotherapy

**DOI:** 10.1186/s13046-018-0756-9

**Published:** 2018-04-24

**Authors:** Branson Chen, Jong Bok Lee, Hyeonjeong Kang, Mark D. Minden, Li Zhang

**Affiliations:** 1 Toronto General Hospital Research Institute, University Health Network, Toronto, Ontario Canada; 2 Department of Laboratory Medicine and Pathobiology, University of Toronto, Toronto, Ontario Canada; 3 Department of Immunology, University of Toronto, Toronto, Ontario Canada; 4 Princess Margaret Cancer Centre, University Health Network, Toronto, Ontario Canada

**Keywords:** Allogeneic double negative T cell, Acute myeloid leukemia, Chemotherapy, Adoptive cellular therapy

## Abstract

**Background:**

While conventional chemotherapy is effective at eliminating the bulk of leukemic cells, chemotherapy resistance in acute myeloid leukemia (AML) is a prevalent problem that hinders conventional therapies and contributes to disease relapse, and ultimately patient death. We have recently shown that allogeneic double negative T cells (DNTs) are able to target the majority of primary AML blasts *in vitro* and in patient-derived xenograft models. However, some primary AML blast samples are resistant to DNT cell therapy. Given the differences in the modes of action of DNTs and chemotherapy, we hypothesize that DNT therapy can be used in combination with conventional chemotherapy to further improve their anti-leukemic effects and to target chemotherapy-resistant disease.

**Methods:**

Drug titration assays and flow-based cytotoxicity assays using *ex vivo* expanded allogeneic DNTs were performed on multiple AML cell lines to identify therapy-resistance. Primary AML samples were also tested to validate our *in vitro* findings. Further, a xenograft model was employed to demonstrate the feasibility of combining conventional chemotherapy and adoptive DNT therapy to target therapy-resistant AML. Lastly, blocking assays with neutralizing antibodies were employed to determine the mechanism by which chemotherapy increases the susceptibility of AML to DNT-mediated cytotoxicity.

**Results:**

Here, we demonstrate that KG1a, a stem-like AML cell line that is resistant to DNTs and chemotherapy, and chemotherapy-resistant primary AML samples both became more susceptible to DNT-mediated cytotoxicity *in vitro* following pre-treatment with daunorubicin. Moreover, chemotherapy treatment followed by adoptive DNT cell therapy significantly decreased bone marrow engraftment of KG1a in a xenograft model. Mechanistically, daunorubicin increased the expression of NKG2D and DNAM-1 ligands on KG1a; blocking of these pathways attenuated DNT-mediated cytotoxicity.

**Conclusions:**

Our results demonstrate the feasibility and benefit of using DNTs as an immunotherapy after the administration of conventional chemotherapy.

**Electronic supplementary material:**

The online version of this article (10.1186/s13046-018-0756-9) contains supplementary material, which is available to authorized users.

## Background

Acute myeloid leukemia (AML) is the most common form of acute leukemia in adults [1] with only ~ 20% of patients expected to survive past 5 years after diagnosis [2]. Despite significant advances in the field of AML pathophysiology, only a few novel therapies for AML have moved into the clinic for a subset of AML cases; consequently, AML relapse remains a significant issue that adversely impacts AML patient survival [3–6]. Cytarabine (AraC) and daunorubicin (DNR) are conventional chemotherapy drugs widely used over the past three decades for induction therapy, which aims to eliminate the bulk of AML blasts by targeting rapidly proliferating cancer cells. Many AML patients achieve initial remission and will receive consolidation therapy, such as high-dose AraC in order to target the remaining AML blasts. Unfortunately, these therapeutic regimens are extremely intensive and toxic [7–9], making them unfeasible for debilitated elderly patients. Despite the improved prognostic information obtained from identifying key cytogenetic and molecular abnormalities to help guide treatment selection, progress on new treatments has not advanced as much as our understanding of the factors that drive the disease [10, 11]. Thus, new therapeutic strategies with lower toxicities are needed to effectively eliminate chemotherapy-resistant AML so as to improve patient survival.

Donor T cells can effectively target AML cells, as evidenced by the strong and curative graft-versus-leukemia effects following allogeneic hematopoietic stem cell transplantation (alloHSCT) or occasionally following donor lymphocyte infusions, which help prevent disease relapse and increase disease-free survival rate [12–14]. T cell-based therapies have therefore been viewed as having potential in curing AML by targeting relapse-initiating AML. However, alloHSCT and donor lymphocyte infusions have a significant drawback in that they can also cause crippling graft versus host disease (GvHD), where the activity of donor cells against host cells is not limited to transformed cells [13, 15].

Our lab was the first to identify CD4 and CD8 double negative T cells (DNTs) in mice [16] and demonstrate the anti-leukemic effects of their *ex vivo* expanded human counterpart *in vitro* and in vivo [17, 18]. We showed that *ex vivo* expanded allogeneic human DNTs can selectively target AML cells, including those obtained from chemotherapy-resistant patients, without causing toxicity towards normal cells and tissues in an in vivo mouse model [18]. Accordingly, a first-in-human phase I clinical trial using allogeneic DNTs to treat patients with high-risk AML has been initiated (NCT03027102). Although DNTs target a wide range of primary AML samples, blasts from approximately 22% of AML patients are not sensitive to DNT-mediated cytotoxicity *in vitro*. Furthermore, administering DNTs as a stand-alone therapy is not curative in patient-derived xenograft models [18].

Induction chemotherapy is administered to most AML patients with curative intent; there is increasing evidence that the cures are in part due to enhanced anti-tumor immune responses [19–21]. Given this, it is reasonable to explore combining standard of care chemotherapy with immune-mediated killing. To the best of our knowledge, there are no reports of combining conventional chemotherapy with adoptive T cell therapy against AML in a xenograft model. Given that DNTs have the potential to be used as an off-the-shelf adjuvant cellular therapy due to their non-HLA-restricted, non-TCR-dependent mode of action [18] and ability to broadly target AML cells from some, but not all chemotherapy-resistant patients, it is of interest to know whether conventional chemotherapy would increase the effectiveness of DNTs against chemotherapy-resistant forms of AML. Furthermore, since about 30% of AML patients do not respond to conventional chemotherapy and a significant portion of their AML cells can be targeted by DNTs [18], it is important to know whether DNT therapy would be complementary to conventional chemotherapy to increase response rate and survival.

## Methods

### Human samples and cell lines

Human myeloid leukemia cell lines OCI-AML-2, OCI-AML-3, KG1a, and MV4–11 were obtained from ATCC. AML2 and AML3 were cultured in alpha-MEM supplemented with 10% fetal bovine serum (FBS), KG1a was cultured in RPMI-1640 supplemented with 10% FBS and MV4–11 was cultured in IMDM supplemented with 10% FBS. All cell lines were incubated at 37 °C in 5% CO_2_. Human blood samples were obtained from healthy adult donors and AML patients, respectively, after obtaining written informed consent and were used according to University Health Network (UHN) Research Ethics Board (05–0221-T) and NHLBI approved protocols. Peripheral blood mononuclear cells (PBMCs) from healthy donors (HDs) or AML patients were separated by Ficoll (GE Healthcare) density gradient. AML patient samples were viably frozen in 10% DMSO, 40% fetal calf serum (FCS) and alpha MEM at the Princess Margaret Leukemia Bank and stored in the vapor phase of liquid nitrogen until used.

### Chemotherapy drugs and treatment

Chemotherapy drugs AraC and DNR (Sigma-Aldrich) were reconstituted in 0.2 μm filtered water and stored in aliquots at − 20 °C. Chemotherapy was added to target cells for 24 h, then incubated at 37 °C in 5% CO_2_. The cells were then washed with RPMI-1640 before use in experiments.

### Ex vivo expansion of human DNTs

Peripheral blood samples were obtained from healthy donors under a UHN-REB approved protocol (05–0221-T). DNTs were enriched from the whole blood by using CD4 and CD8 RosetteSep depletion kits according to the manufacturer’s instructions (StemCell Technologies). The samples were then layered on Ficoll-Paque (GE Healthcare) and centrifuged at 1200 x g for 20 min. The enriched DNTs were expanded *ex vivo* as described previously [17]. DNTs from d12 to d20 of culture were used in experiments.

### Flow cytometry

The following anti-human antibodies for staining of cell surface markers were used: CD3 (HIT3a), CD33 (WM53), CD45 (HI30), CD34 (561), CD112 (TX31), CD155 (SKII.4), MIC-A/B (6D4), Annexin V, and 7AAD, which were all purchased from BioLegend, and ULBP4 (709116) from R & D Systems. Data acquisition was performed using C6 Accuri (BD Biosciences), LSRII (BD Biosciences), or Attune NxT (ThermoFisher) flow cytometers and data were analyzed using FlowJo version 10.

### Cytotoxicity assays and blocking experiments

The cytotoxic activity of DNTs was measured by a 2 h or 4 h flow-based killing assay. Target cells were labelled with PKH-26 (Sigma-Aldrich) according to the manufacturer’s instructions, and then co-incubated with DNTs at appropriate effector to target (E:T) ratios in U-bottom 96-well plates (Corning). Dead cells were identified as the PKH^+^CD3^−^AnnexinV^+^ by flow cytometry. Gating strategies for patient leukemic blasts varied according to the phenotype of the AML cells. Percent specific killing was calculated using the formula:

$$ \% Specific\kern0.5em Killing\kern0.5em =\frac{\left(\%{AnnexinV}_{with\kern0.5em DNT}-\%{AnnexinV}_{Without\kern0.5em DNT}\right)}{\left(100\%-\%{AnnexinV}_{with out\kern0.5em DNT}\right)}\times 100\kern0.5em \% $$

Blocking antibodies for NKG2D and DNAM-1 (CD226), or the isotype control (BioLegend) were incubated with DNTs at a final concentration of 10 μg/mL for 30 min and washed before co-incubation with target cells.

### Xenograft models

NOD.Cg-*Prkdc*^*scid*^ *Il2rg*^*tm1Wjl*^/SzJ (NSG) mice (Jackson Laboratories) were maintained at UHN animal facility in accordance with the guidelines of the Animal Care Committee of UHN and the Canadian Council on Animal Care. On day 0, 8- to 12-week-old female NSG mice were irradiated (225 cGy) and then injected with 4 × 10^6^ KG1a cells intravenously (i.v.). On day 5, mice were administered a “5 + 3” chemotherapy regimen as described by Wunderlich et al. [22], but at an adjusted lower dose (8 mg/kg AraC + 0.24 mg/kg DNR). 20 × 10^6^ DNTs were then injected i.v. on days 12, 15, and 18. rIL2 (Proleukin, 10^4^ IU/mouse) was administered i.v. along with DNT infusions and was also given intraperitoneally on days 21, 24, and 27. Mice were sacrificed 6 weeks after engraftment of KG1a and bone marrows were harvested and processed using standard techniques. Leukemic engraftment was determined by flow cytometry gating on the human CD45^+^CD34^+^ population.

### Statistical analysis

Statistical analyses were performed using GraphPad Prism version 6 (San Diego, CA, USA). Data were expressed as means + standard deviation (SD). Two-tailed unpaired or paired Student *t* tests, one-way ANOVAs with Newman-Keul multiple comparisons test correction, and repeated measures ANOVAs with Holm-Sidak’s multiple comparisons test correction were performed, where appropriate, to identify significant differences between groups in our experiments.

## Results

### Stem-like AML cell line KG1a is resistant to both chemotherapy and DNT-mediated cytotoxicity

**Fig. 1:**
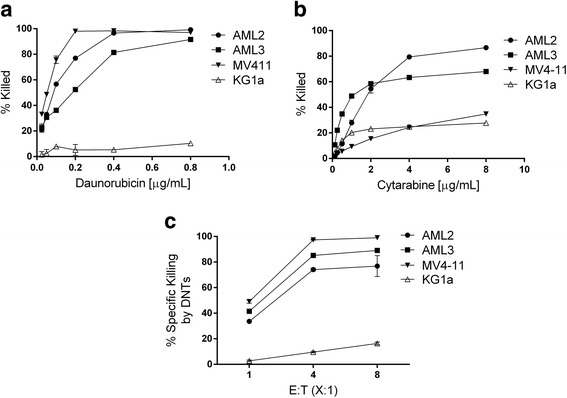
KG1a is resistant to chemotherapy and DNT therapy. **a** A dose titration of DNR, ranging from 0.025 to 0.8 μg/mL, and **b** AraC, ranging from 0.125 to 8 μg/mL, were performed on four different AML cell lines over 24 h and the proportion of cells killed by the drugs, determined by flow cytometry, is shown. **c** Cytotoxicity assays were performed on four different AML cell lines, which were co-cultured with DNTs for 2 h at varying effector-to-target (E:T) ratios. Specific killing of target cells by DNTs was calculated as described in the Methods section. Each point represents the mean + standard deviation (SD) of triplicate measurements from a representative experiment. Experiments were repeated twice with similar results

The first-line “7 + 3” induction therapy with AraC and DNR is the most commonly-used regimen in treating AML [23]. We first wanted to identify AML cell lines that are relatively resistant to chemotherapy and DNT cell-mediated cytotoxicity. We determined the susceptibility of OCI-AML2 (AML2), OCI-AML3 (AML3), MV4–11, and KG1a to these conventional chemotherapy drugs via a titration of the drugs in an overnight assay. We observe that KG1a, a CD34^+^ AML cell line described to be stem-like [24], was noticeably more resistant to DNR when compared to the other AML cell lines (fig. 1a). When treated with 0.8 μg/mL of DNR, only 10% of KG1a cells were killed, whereas > 90% of the other three AML cell lines were killed (Fig. 1a). The four AML cell lines had varying susceptibility to AraC, but at the highest dose we tested, KG1a was also the most resistant to this drug compared to the other cell lines (Fig. 1b). Next, we tested the susceptibility of the four AML cell lines to DNT-mediated cytotoxicity using a flow cytometry-based cytotoxicity assay [18]. We observed in AML2, AML3, and MV4–11, that > 70% of the cells were killed by DNTs at a 4:1 effector to target (E:T) ratio after a 2 h co-incubation (Fig. 1c). KG1a, on the other hand, was less susceptible to DNTs in comparison to the other lines, with only 10% being killed by DNTs under the same condition (Fig. 1c). Specific cytotoxicity of KG1a still remained below 30% even after 24 h of co-culture, whereas the three other AML cell lines were almost completely killed (data not shown). Representative flow plots and gating strategies for the cytotoxicity assays are shown in (Additional file 1 Figure S1). These data demonstrate that the stem-like AML cell line KG1a is resistant to both chemotherapy and DNTs.

### Chemotherapy increases the sensitivity of leukemic cell lines to DNT-mediated cytotoxicity

**Fig. 2:**
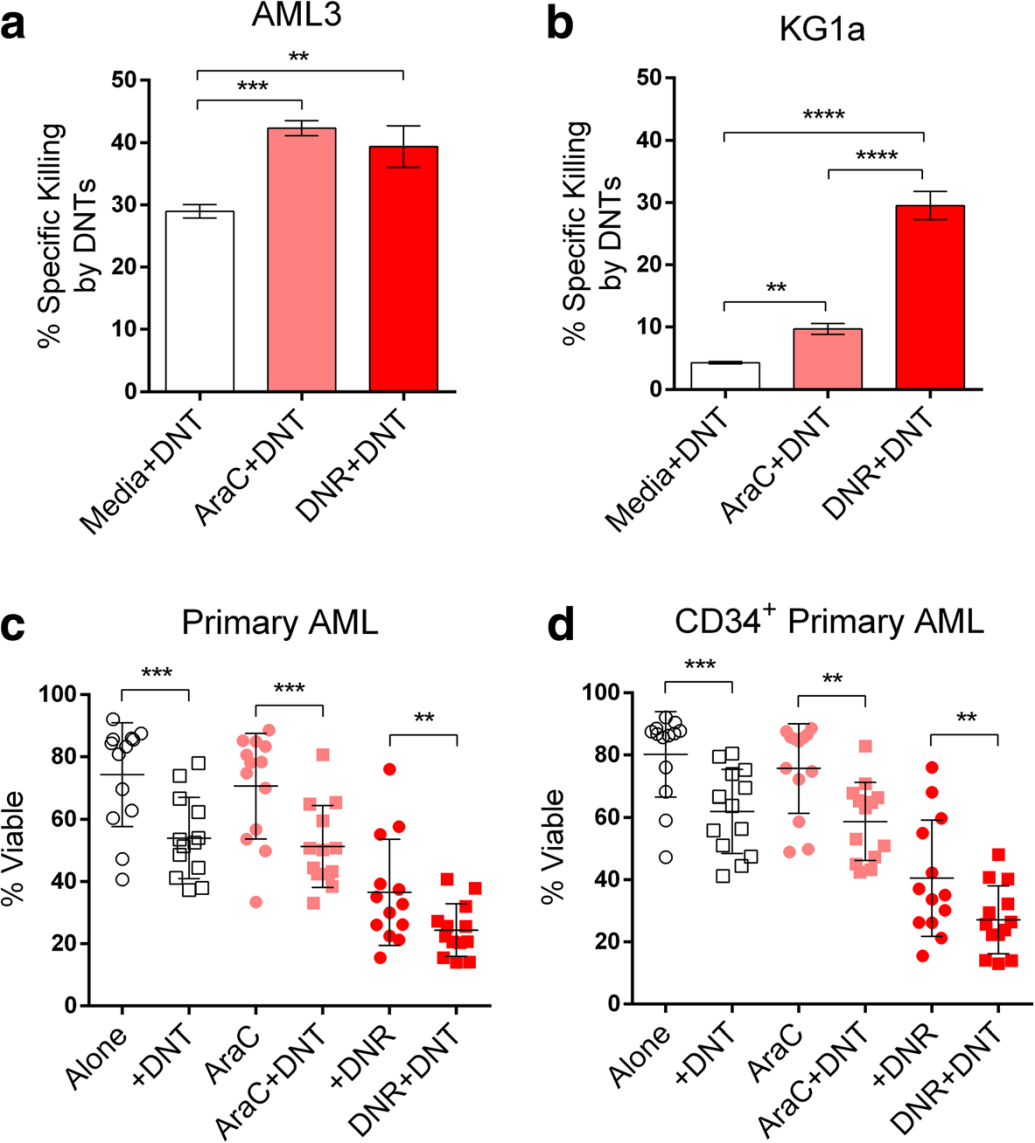
Chemotherapy pre-treatment sensitizes AML cells to DNT-mediated cytotoxicity. **a** AML3 and **b** KG1a cells were treated with media, 0.25 μg/mL AraC, or 0.4 μg/mL DNR for 24 h before co-culture with DNTs at a 1:1 or 4:1 E:T ratio, respectively. % Specific killing by DNTs was measured by the flow-based killing assay as described in the Methods section. These experiments were repeated 3 times with similar results. **c** & **d** Primary AML blasts collected from 13 AML patients were cultured for 24 h in complete media and either 0.25 μg/mL AraC, or 0.4 μg/mL DNR, followed by a 2 h incubation with DNTs at an E:T ratio of 2:1. Percentages of viable **c** AML cells or **d** CD34^+^ AML cells were determined by flow cytometry analysis. **, *p* < 0.01; ***, *p* < 0.001; ****, *p* < 0.0001

In order to determine whether the use of DNTs in combination with standard chemotherapy produces an enhanced anti-leukemic effect, we pre-treated AML cells with chemotherapy for 24 h, prior to their co-culture with DNTs. The assay conducted and the calculation of specific killing are illustrated in (Additional File 2 Figure S2). The concentrations of AraC and DNR used in our *in vitro* experiments were within the ranges of normal plasma concentrations of AraC (up to 0.41 μg/mL) and DNR (up to 0.74 μg/mL) in AML patients after infusion [25, 26]. Both AML3 (Fig. 2a) and KG1a (Fig. 2b) became significantly more susceptible to DNT-mediated cytotoxicity after chemotherapy pre-treatment. Specific killing of AML3 by DNTs following AraC (42.34 ± 1.21%) and DNR (39.40 ± 3.34%) pre-treatment was significantly higher compared to treatment with the vehicle control (28.96 ± 1.08%) (Fig. 2a). Notably, we observed specific killing of KG1a by DNTs to be greatly enhanced after DNR pre-treatment (29.54 ± 2.26%) compared to the vehicle control (4.33 ± 0.18%). There was a significant, albeit smaller, effect with AraC (9.69 ± 0.88%) (Fig. 2b); the original percentage of dead cells are shown in (Additional file 3 Table S1). Of note, the combination of AraC and DNR at the optimal molar ratio [27, 28] did not produce an additive effect on DNT cytotoxicity (Additional file 4 Figure S3). These experiments show that conventional chemotherapy was able to increase the sensitivity of AML cells to DNT cell-mediated cytotoxicity.

### DNTs further reduce the viability of primary AML blasts after chemotherapy pre-treatment

To validate the results obtained using AML cell lines, primary AML samples were obtained from 13 patients (Additional file 5 Table S2) and pretreated with DNR or AraC followed by co-incubation with DNTs. In the absence of any treatment, primary AML cells were 74.33 ± 16.62% viable and DNTs were able to reduce their viability to 53.99 ± 13.00% (Fig. 2c). Furthermore, DNTs exhibited the capacity to further reduce the percentage of viable primary AML cells even after AraC (from 70.61 ± 16.91% to 51.27 ± 13.17%) or DNR (from 36.58 ± 17.09% to 24.40 ± 8.46%) pre-treatment (Fig. 2c). Since CD34^+^ populations are enriched for progenitor cells [29, 30], characterized as being apoptosis-resistant [31], and known to be a marker for poor prognosis [32, 33], we also specifically examined this population by gating on CD34^+^ target cells. We observed a similar ability of DNTs to further target CD34^+^ primary blasts after chemotherapy treatment (Fig. 2d). These data demonstrate the ability of DNTs to have cytotoxic effects on the remaining viable CD34^+^ primary AML cells after chemotherapy treatment.

### Chemotherapy and DNT combination therapy effectively reduce engraftment of KG1a in a mouse model

**Fig. 3:**
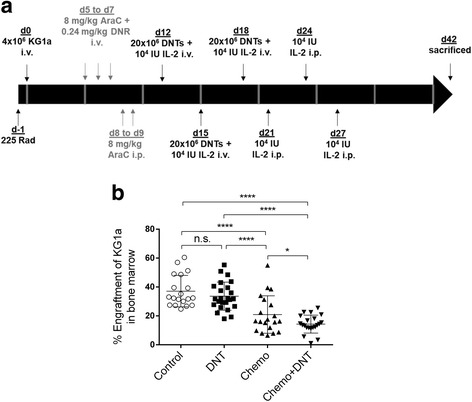
Chemotherapy-DNT combination therapy reduces KG1a engraftment in vivo. **a** The study protocol for the chemotherapy-DNT combination therapy in NSG mice. **b** A summary of 3 independently performed experiments (*n* = 5–10/group per experiment), where NSG mice were engrafted with 4 million KG1a cells i.v., followed by DNT therapy alone, chemotherapy alone, or a combination of the two therapies. Mice were sacrificed 6 weeks post-engraftment for analysis of KG1a engraftment in the bone marrow by gating on human CD45^+^CD34^+^ cells and analyzed by flow cytometry. *, *p* < 0.05; ****, p < 0.0001

Few studies have examined the effect of adoptive T cell therapy against AML in vivo, and no studies have tested the combination of chemotherapy and adoptive T cell therapy in an AML xenograft model thus far. To determine whether combining adoptive transfer of DNTs with low-dose chemotherapy can effectively target KG1a, which is resistant to both chemotherapy and DNTs *in vitro* (fig. 1)*,* we first titrated a chemotherapy regimen based on the report by Wunderlich et al. [22] to a tolerable, significantly lower level as shown in (Additional file 6 Figure S4). Next, NSG mice were injected with KG1a cells and treated with the low-dose (8 mg/kg AraC + 0.24 mg/kg DNR, or 16% of maximum tolerated dose) chemotherapy or a vehicle control, with or without DNT therapy as schematically shown in Fig. 3a. Consistent with our *in vitro* findings, DNT treatment alone did not significantly reduce leukemia cell engraftment in the bone marrow of the recipient mice (Fig. 3b). Yet, while KG1a was resistant to either AraC or DNR *in vitro*, the combination of both chemotherapeutics significantly reduced the engraftment of KG1a in vivo. This anti-leukemic effect was further enhanced by additional treatment with adoptive transfer of DNTs. Mice treated with the combination therapy had significantly lower bone marrow engraftment of KG1a compared to the chemotherapy treatment alone (Fig. 3b). Representative flow plots and gating strategies are shown in (Additional File 7 Figure S5).

### Daunorubicin (DNR) sensitizes some primary AML patient samples to DNTs *in vitro*

**Fig. 4:**
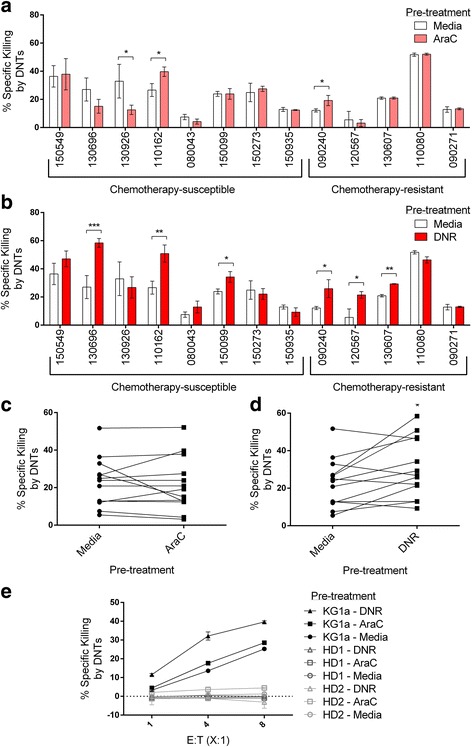
CD34^+^ primary AML blasts become sensitized to DNT-mediated cytotoxicity after daunorubicin pre-treatment. **a** & **b** Primary AML blasts from patients were grouped according to whether the patients had undergone complete remission (chemotherapy-susceptible, *n* = 8) or were relapse/resistant (chemotherapy-resistant, n = 5). Primary AML blasts were cultured for 24 h in complete media and either **a** 0.25 μg/mL AraC, or **b** 0.4 μg/mL DNR, followed by a 2 h incubation with DNTs at an E:T ratio of 2:1. % Specific killing by DNTs after chemotherapy pre-treatment is shown in comparison to the vehicle control as pre-treatment. **c** & **d** Averages from Figs. 4a and b were plotted in pairs (each pair represents an individual patient) to compare the overall effect of **c**) AraC or **d**) DNR on the susceptibility of primary AML samples to DNTs. **e** Normal PBMCs (*n* = 2), obtained from healthy donors (HDs), along with KG1a were pre-treated with AraC, DNR, or the vehicle control, followed by a co-culture with DNTs at varying E:T ratios. All of the cytotoxicity assays were performed using DNTs that were allogeneic to the patient or HD samples. *, *p* < 0.05; **, *p* < 0.01; ***, *p* < 0.001.

Although DNTs can further decrease the viability of CD34^+^ primary AML samples after chemotherapy pre-treatment, as shown in Fig. 2d, we wanted to determine if chemotherapy in fact sensitizes the samples to greater DNT-mediated killing. To this end, we analyzed % specific killing of the primary AML samples by DNTs after chemotherapy treatment compared to after a vehicle control. AraC pre-treatment only sensitized 2/13 primary AML samples to DNT-mediated cytotoxicity (Fig. 4a). DNR pre-treatment, on the other hand, was able to increase the susceptibility of approximately half of the primary AML samples (3/8 chemotherapy-susceptible and 3/5 chemotherapy-resistant) to DNT-mediated cytotoxicity (Fig. 4b). When analyzing the entire population of primary samples, the difference in mean % specific killing was not significantly different after AraC pre-treatment (Fig. 4c, *p* = 0.66), but significantly higher after DNR pre-treatment (Fig. 4d, *p* = 0.03). Furthermore, we previously showed that DNTs do not cause GvHD in a xenogeneic model nor target normal PBMCs *in vitro* [18]. When PBMCs obtained from healthy donors (HDs) were treated with chemotherapy followed by co-incubation with DNTs, there was no observed cytotoxicity to normal PBMCs (Fig. 4e), indicating that chemotherapy treatment of normal PBMCs does not sensitize them to DNT-mediated cytotoxicity.

### Chemotherapy enhances sensitivity of KG1a to DNTs by an increase in NKG2D/DNAM-1 signaling

**Fig. 5:**
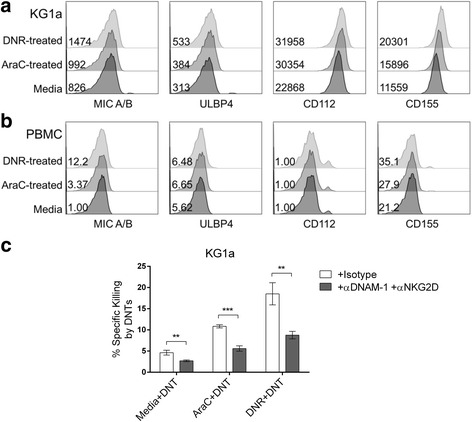
NKG2D and DNAM-1 play a role in chemotherapy-induced sensitivity to DNTs. **a** & **b** Representative histograms of the expression of NKG2D ligands (MIC-A/B, ULBP4) and DNAM-1 ligands (CD112, CD155) are shown. **a** KG1a or **b** PBMCs from an HD were treated with media (dark grey), AraC (grey), and DNR (light grey) and then analysed for surface expression of NKG2D and DNAM-1 ligands by flow cytometry. Bolded numbers represent the mean fluorescence intensity of each stain. **c** KG1a were treated with 0.25 μg/mL AraC or 0.4 μg/mL DNR for 24 h and used as targets in a blocking assay. DNTs were incubated with anti-NKG2D (10 μg/mL) and anti-DNAM-1 (10 μg/mL) or 20 μg/mL of the isotype for 30 min and then washed with media prior to co-culture with chemotherapy pre-treated KG1a at an E:T ratio of 4:1. These experiments were conducted independently 3 times. **, *p* < 0.01; ***, *p* < 0.001

Cancer cells can be sensitized to immune cell lysis by chemotherapy through the upregulation of natural-killer group 2, member D (NKG2D) ligands [34]. There is also evidence that chemotherapy can induce the expression of NKG2D or DNAX Accessory Molecule-1 (DNAM-1) ligands on cancer cells [35]. In the same vein, we observed an increased expression of NKG2D ligands and DNAM-1 ligands on KG1a following a 24 h chemotherapy treatment. We consistently saw increases in the mean fluorescence intensity (MFI) of these ligands after chemotherapy treatment compared to a media control, with DNR treatment having a greater effect than AraC (Fig. 5a). We did not observe a noticeable increase in the expression of the same ligands on PBMCs from healthy donors after treatment with either chemotherapy drugs, however (Fig. 5b). Next, to determine the involvement of these pathways in the targeting of chemotherapy-treated KG1a by DNTs, we performed blocking assays using neutralizing antibodies against the two receptors. Anti-NKG2D and anti-DNAM-1 neutralizing antibodies were added to DNTs, with subsequent reduced specific killing of KG1a and chemotherapy-treated KG1a (Fig. 5c). Despite this, we saw the greatest decrease with DNR-treated KG1a (18.52 ± 2.62% to 8.77 ± 0.88%). These data indicate that chemotherapy can increase DNT-mediated cytotoxicity at least partially through upregulation of NKG2D and DNAM-1 ligands expression.

## Discussion

We previously demonstrated the feasibility of expanding therapeutic quality and quantity of DNTs and the capabilities of DNTs against AML, among other forms of leukemia and lymphoma [18]. Herein we explored the use of DNTs in a combinatorial approach with conventional chemotherapy against chemotherapy-resistant AML. Using KG1a, an AML cell line that is resistant to NK cell lysis and chemotherapy [24], and CD34^+^ primary AML samples, which are resistant to apoptosis [31], the data presented further supports the effectiveness of DNTs against therapy-resistant cells. Moreover, these results show that prior treatment with chemotherapy such as DNR sensitizes AML cells to DNT killing.

KG1a is resistant *in vitro* to apoptosis induced either by chemotherapy (Figs. 1a and b) or DNT-mediated cytotoxicity (Fig. 1c). We also found that the cell line is resistant to DNT therapy in vivo (Fig. 3b). However, our results show that engraftment of KG1a in the bone marrow can be significantly reduced by chemotherapy in vivo (Fig. 3b). This may be due to administering a 5-day regimen that consists of both chemotherapy drugs, compared to when single chemotherapy drugs were added for 24 h *in vitro*. The pharmacokinetics and therapeutic effects of the drugs in vivo over a prolonged period would conceivably be different from a 24 h *in vitro* treatment, and the combination of the two drugs may have additive or synergistic effects in targeting KG1a. A previous report also described similar characteristics of this cell line *in vitro* [24]. Importantly, despite the resistance of KG1a to conventional therapies relative to other AML lines, a greater anti-leukemic effect was observed both *in vitro* (Fig. 2b) and in vivo (Fig. 3b) when we used a combinatorial approach. Furthermore, we examined, through cytotoxicity assays, the effectiveness of the combination therapy on CD34^+^ primary AML samples. Most notably, we observed a significant increase in specific killing of these cells by DNTs after pre-treatment with DNR in approximately half (6/13) of the samples (Fig. 4b). Similar to what we demonstrated with KG1a (Fig. 2b), AraC pre-treatment did not elicit a sensitizing effect comparable to that of DNR (Figs. 4a and c). These observations are in keeping with reports in the literature that the family of chemotherapy drugs encompassing DNR is known to elicit immunogenic cell death by calreticulin translocation and the release of high-mobility-group box 1 [36].

AML is known to be an extremely heterogeneous disease; this is reflected in our finding that some of the primary AML samples appeared to be targeted more effectively by DNTs than others after chemotherapy (Figs. 4a and b). The % specific killing calculation (see Additional file 2 Figure S2) takes into account the spontaneous and chemotherapy-induced cell death to ultimately determine the proportion of cells that are solely targeted by DNTs. Since we detected specific killing of all primary AML samples by DNTs, it is expected that combining DNTs and chemotherapy can target more AML cells than chemotherapy alone. Accordingly, we saw the effect of DNTs in reducing the proportion of viable AML blasts *in vitro* after chemotherapy treatment (Figs. 2c and d). Nevertheless, 1 out of 13 primary AML samples became significantly less sensitive to DNTs after AraC treatment (Fig. 4a). To circumvent the issue of potential antagonism between the two therapies, pre-screening patients after they have undergone chemotherapy to determine the sensitivity of their AML cells to DNTs *in vitro* may help to stratify patient selection or regimen.

In our in vivo experiments, mice were administered a “5 + 3” chemotherapy regimen as described by Wunderlich et al. [22], but at an adjusted lower dose (8 mg/kg AraC + 0.24 mg/kg DNR), which we established through titrating the drugs in vivo (see Additional File 6 Figure S4). Our in vivo studies demonstrated that DNT therapy alone was ineffective at reducing engraftment of KG1a. While low-dose chemotherapy treatment significantly reduced KG1a engraftment in the bone marrow, we observed an even greater reduction with the combination of DNT therapy and chemotherapy (Fig. 3b). In the clinic, almost all AML patients receive chemotherapy, which is effective in reducing the bulk of AML cells. Since our *in vitro* and in vivo data indicate that chemotherapy can also prime the remaining AML blasts to be more susceptible to DNT-mediated cytotoxicity, it suggests that DNTs can be used as an adjuvant and administered shortly after chemotherapy in order to take advantage of the sensitizing effects of chemotherapy to eliminate chemotherapy-resistant residual AML cells. Based on our model using a reduced chemotherapy dose, which was 16% of the maximum tolerated dose in NSG mice [22], perhaps a lower dose can be used in clinic as well when combined with DNT therapy, in the hope of reducing the various side-effects and toxicities of chemotherapy. This would greatly benefit elderly patients, who have much poorer prognosis than the rest of the population and have additional risk factors that prevent them from being eligible for remission style therapy [37–39]. Additionally, there are current efforts by others to optimize conventional chemotherapy drug delivery in AML patients to reduce toxicities [40], which have led to a phase III clinical trial of CPX-351, using a liposomal formulation of daunorubicin and cytarabine to treat elderly patients with high risk (secondary) AML (NCT01696084). The advent of newer technologies that can more efficiently administer chemotherapeutics to patients while avoiding side-effects can pave the way for more effective combination therapies.

Chemotherapeutic agents are known to influence our immune system in various ways [19]. Specifically, chemotherapeutics can induce expression of various markers on the surface of cancer cells to facilitate their lysis by cytotoxic immune cells or induce the release of soluble factors that in turn stimulate immune responses [20, 21]. There is also evidence that anthracyclines, a family of chemotherapy drugs that DNR is part of, have strong, immunogenic effects [36]. The role of NKG2D and DNAM-1 receptor-ligand interactions in cell-based immunotherapies is well-described [41, 42]. Likewise, the blocking experiments in this study demonstrated a role of NKG2D and DNAM-1 on DNTs in the targeting of chemotherapy-treated KG1a (Fig. 5c). We also observed the ability of DNR and, to a lesser extent, AraC, to increase the expression of NKG2D and DNAM-1 ligands in KG1a (Fig. 5a). PBMCs from healthy donors, however, did not express nor upregulate the ligands after chemotherapy pre-treatment (Fig. 5b). The blocking assay using anti-NKG2D and anti-DNAM-1 antibodies significantly reduced but did not fully abrogate the targeting of DNR-treated KG1a by DNTs (Fig. 5c), which suggests that other pathways may be involved. As there are many ways that chemotherapy drugs are able to influence the immune system and immune function [19], future studies are required to explore the full range of their immunogenic effects so as to identify other mechanisms involved in the chemotherapy-induced susceptibility of AML cells to DNTs.

## Conclusions

We demonstrate, for the first time in a xenograft model, the effectiveness of combining an adoptive T cell therapy and low-dose chemotherapy in reducing the engraftment of therapy-resistant AML. We also observe complementary activity between conventional chemotherapy and DNT therapy on CD34^+^ primary AML samples, which suggests that DNTs can target chemotherapy-resistant cells in a clinical setting, especially after DNR treatment. Overall, the results of this study support the use of DNTs as an adjuvant cellular therapy following administration of chemotherapy.

## Additional files

Additional file 1: **Figure S1.** Gating strategy for 2 h flow-based cytotoxicity assay. Target cells were stained with PKH-26, according to manufacturer’s instructions and then effector and target cells were cultured together for 2 h before staining was performed. The co-culture was stained for CD3 and Annexin-V. Here we first gated out the debris, removed doublets, gated on the PKH^+^CD3^−^ population and then determined the percentage of Annexin-V+ cells that are undergoing or having undergone apoptosis. (TIF 1571 kb)

Additional file 2: **Figure S2.** Calculation of % specific killing by DNTs post-chemotherapy treatment *in vitro*. This schematic describes the calculations performed to determine % specific killing of target cells by DNTs. Target cells are treated first with chemotherapy drugs or the vehicle control for 24 h and then live cells are counted based on Trypan Blue exclusion. The target cells are then cultured with or without DNTs at the appropriate E:T ratio for the cytotoxicity assay for 2 h. The formula used for % specific killing takes the difference in % of dead AML cells between adding DNTs and the absence of effector cells, and divides this number by the percentage of live cells in the absence of effector cells (taking into account the spontaneous and chemotherapy-induced cell death, irrespective of DNT function). (TIF 1508 kb)

Additional file 3: **Table S1.** Percentages of dead AML3 and KG1a cells after chemotherapy and DNT co-culture. This table shows the original percentages of dead cells in the assays illustrated in Fig. 2A and B, which were performed in triplicate. Flow cytometry analysis, with Annexin-V as a viability marker, was employed to determine cell viability after the treatments. (DOCX 13 kb)

Additional File 4: **Figure S3.** Combining cytarabine and daunorubicin pre-treatment does not elicit additive effects on DNT killing. KG1a cells were treated with media, AraC, DNR, or both drugs for 24 h before co-culture with DNTs at a 4:1 E:T ratio. % Specific killing by DNTs was measured by the flow-based killing assay as described in the Methods section. (TIF 136 kb)

Additional file 5: **Table S2.** Clinical characteristics of 13 AML patients whose AML blasts were used for *in vitro* assays. Abbreviations: WBC – White blood cell; BM – Bone marrow; FAB – The French-American-British classification of AML; MRC – Medical Research Council cytogenetic classification; MDS – Myelodysplastic syndrome; MK – Monosomal Karyotype; NPM1 – Nucleophosmin 1; FLT3-ITD – Fms related tyrosine kinase 3 - internal tandem duplication; FLT3-TKD – Fms related tyrosine kinase 3 - tyrosine kinase domain; BCR-ABL – Break point cluster region - Abelson murine leukemia viral oncogene homolog 1; RAR – Retinoic acid receptor. (DOCX 17 kb)

Additional file 6: **Figure S4.** Chemotherapy dose titration in NSG mice engrafted with KG1a. NSG mice were engrafted with KG1a and a range of chemotherapy doses were tested (*n* = 3/group). We determined a dose (8 mg/kg AraC + 0.24 mg/kg DNR) that would moderately reduce KG1a engraftment in the bone marrow, determined by flow cytometry analysis (described in Methods), while remaining well below the maximum tolerated dose, as determined by Wunderlich et al. [22]. (TIF 188 kb)

Additional file 7: **Figure S5.** Gating strategy for in vivo bone marrow samples. Bone marrows were harvested from KG1a-engrafted mice at 6 weeks and processed using standard techniques. They were then stained with 7AAD (a viability marker) and fluorescently labeled anti-CD45 and anti-CD34. We gated out the debris and red blood cells, removed doublets, gated on live cells, and lastly gated on the human CD45^+^CD34^+^ population to determine the proportion of KG1a cells in the bone marrow. (TIF 1040 kb)

## Abbreviations

AlloHSCT: Allogeneic hematopoietic stem cell transplantation
AML: Acute myeloid leukemia
AraC: Cytarabine
DNAM-1: DNAX accessory molecule 1
DNR: Daunorubicin
DNTs: Double negative T cells
E:T: Effector-to-target
FBS: Fetal bovine serum
GvHD: Graft versus host disease
HDs: Healthy donors
MFI: Mean fluorescence intensity
NKG2D: Natural-killer group 2, member D
NSG: NOD.Cg-Prkdcscid Il2rgtm1Wjl/SzJ
PBMCs: Peripheral blood mononuclear cells
SD: Standard deviation
UHN: University Health Network.
