# Supplementary figures and images for: Targeting chemotherapy-resistant leukemia by combining DNT cellular therapy with conventional chemotherapy
Source: J Exp Clin Cancer Res. 2018 Apr 24;37:88. doi: 10.1186/s13046-018-0756-9 (PMC5916833; doi:10.1186/s13046-018-0756-9)

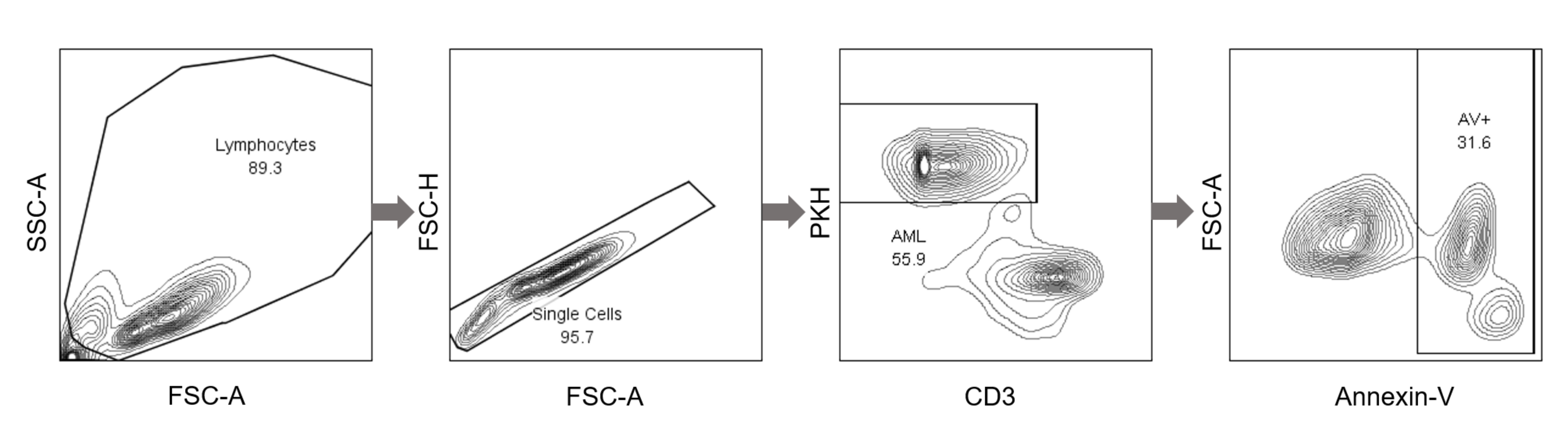

Supplement: Supplementary file 1 — Figure S1. Gating strategy for 2 h flow-based cytotoxicity assay. Target cells were stained with PKH-26, according to manufacturer’s instructions and then effector and target cells were cultured together for 2 h before staining was performed. The co-culture was stained for CD3 and Annexin-V. Here we first gated out the debris, removed doublets, gated on the PKH+CD3− population and then determined the percentage of Annexin-V+ cells that are undergoing or having undergone apoptosis. (TIF 1571 kb) [file 13046_2018_756_MOESM1_ESM.tif]

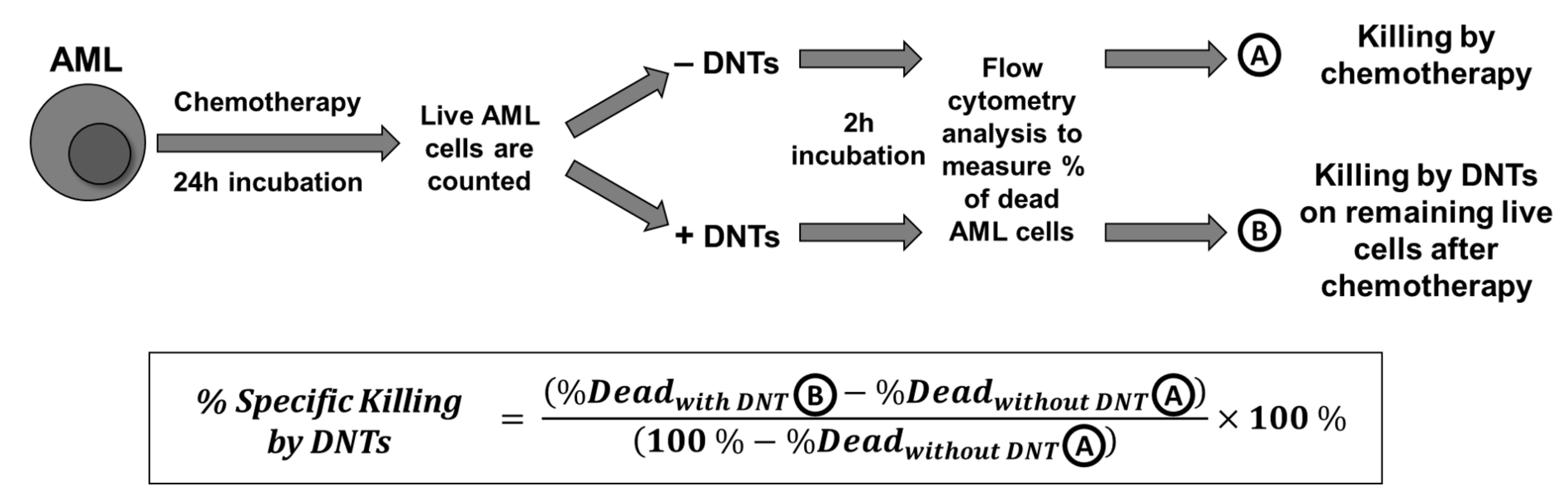

Supplement: Supplementary file 2 — Figure S2. Calculation of % specific killing by DNTs post-chemotherapy treatment in vitro. This schematic describes the calculations performed to determine % specific killing of target cells by DNTs. Target cells are treated first with chemotherapy drugs or the vehicle control for 24 h and then live cells are counted based on Trypan Blue exclusion. The target cells are then cultured with or without DNTs at the appropriate E:T ratio for the cytotoxicity assay for 2 h. The formula used for % specific killing takes the difference in % of dead AML cells between adding DNTs and the absence of effector cells, and divides this number by the percentage of live cells in the absence of effector cells (taking into account the spontaneous and chemotherapy-induced cell death, irrespective of DNT function). (TIF 1508 kb) [file 13046_2018_756_MOESM2_ESM.tif]

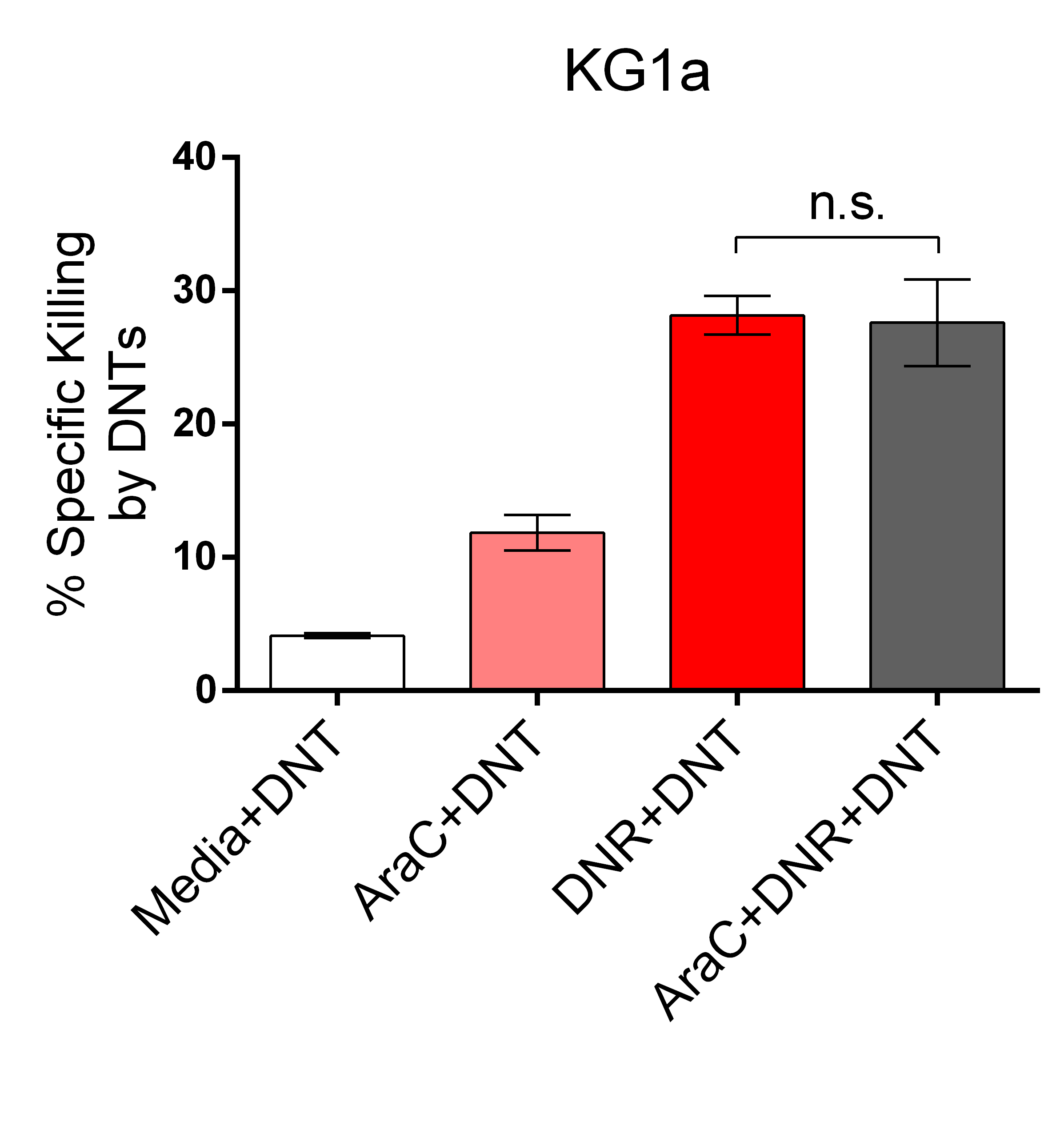

Supplement: Supplementary file 4 — Figure S3. Combining cytarabine and daunorubicin pre-treatment does not elicit additive effects on DNT killing. KG1a cells were treated with media, AraC, DNR, or both drugs for 24 h before co-culture with DNTs at a 4:1 E:T ratio. % Specific killing by DNTs was measured by the flow-based killing assay as described in the Methods section. (TIF 136 kb) [file 13046_2018_756_MOESM4_ESM.tif]

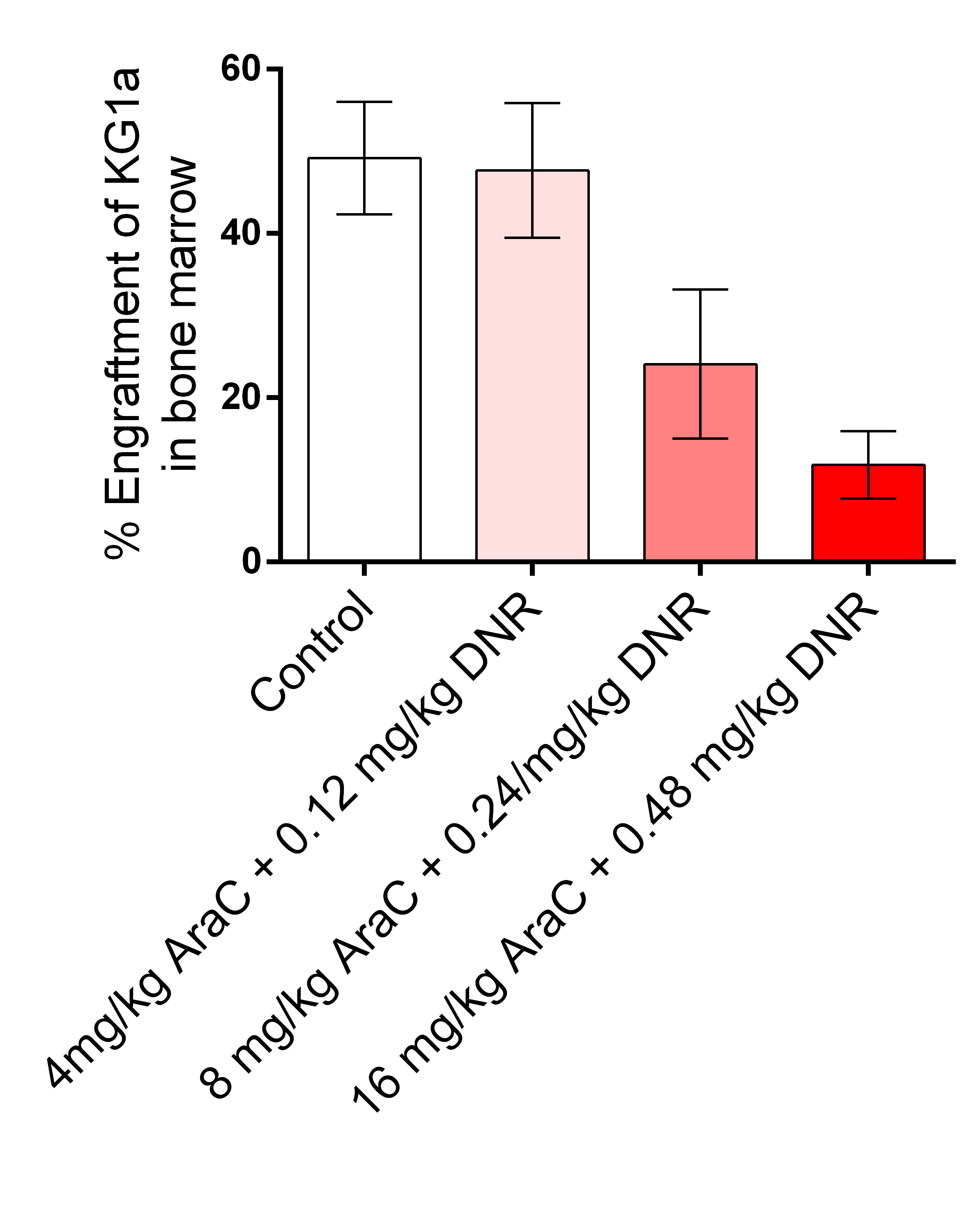

Supplement: Supplementary file 6 — Figure S4. Chemotherapy dose titration in NSG mice engrafted with KG1a. NSG mice were engrafted with KG1a and a range of chemotherapy doses were tested (n = 3/group). We determined a dose (8 mg/kg AraC + 0.24 mg/kg DNR) that would moderately reduce KG1a engraftment in the bone marrow, determined by flow cytometry analysis (described in Methods), while remaining well below the maximum tolerated dose, as determined by Wunderlich et al. [22]. (TIF 188 kb) [file 13046_2018_756_MOESM6_ESM.tif]

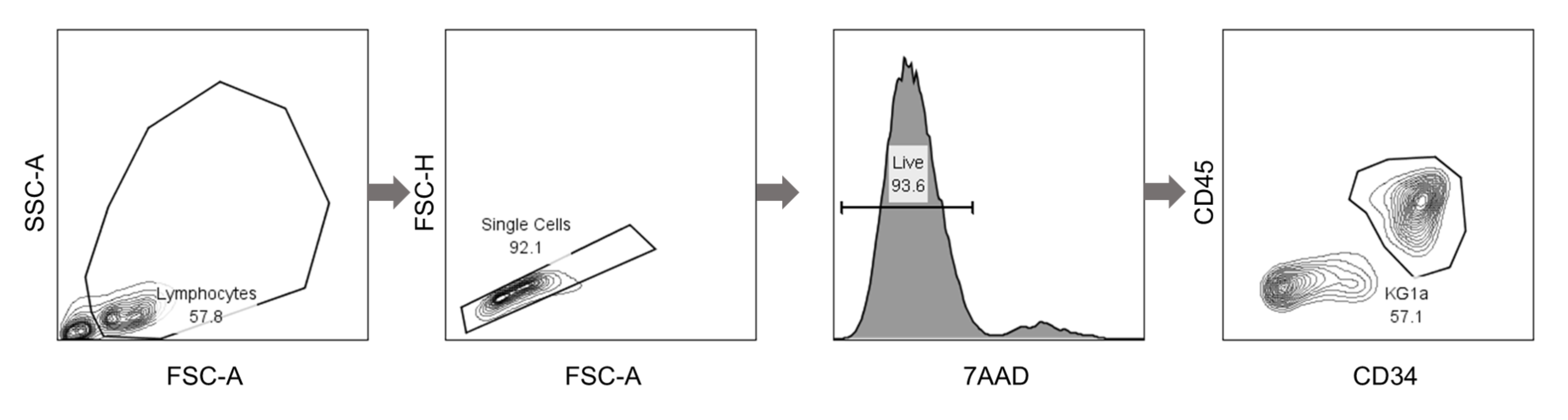

Supplement: Supplementary file 7 — Figure S5. Gating strategy for in vivo bone marrow samples. Bone marrows were harvested from KG1a-engrafted mice at 6 weeks and processed using standard techniques. They were then stained with 7AAD (a viability marker) and fluorescently labeled anti-CD45 and anti-CD34. We gated out the debris and red blood cells, removed doublets, gated on live cells, and lastly gated on the human CD45+CD34+ population to determine the proportion of KG1a cells in the bone marrow. (TIF 1040 kb) [file 13046_2018_756_MOESM7_ESM.tif]
