# Supplementary material for: Targeting chemotherapy-resistant leukemia by combining DNT cellular therapy with conventional chemotherapy
Source: J Exp Clin Cancer Res. 2018 Apr 24;37:88. doi: 10.1186/s13046-018-0756-9 (PMC5916833; doi:10.1186/s13046-018-0756-9)
Supplement: Supplementary file 3 — Table S1. Percentages of dead AML3 and KG1a cells after chemotherapy and DNT co-culture. This table shows the original percentages of dead cells in the assays illustrated in Fig. 2A and B, which were performed in triplicate. Flow cytometry analysis, with Annexin-V as a viability marker, was employed to determine cell viability after the treatments. (DOCX 13 kb) [file 13046_2018_756_MOESM3_ESM.docx]

| **Proportion of dead AML3 (Figure 2A)** | | | | **Proportion of dead KG1a (Figure 2B)** | | | |
| --- | --- | --- | --- | --- | --- | --- | --- |
|  | Media | AraC | DNR |  | Media | AraC | DNR |
| w/o DNT | 5.28% | 31.1% | 18.9% | w/o DNT | 1.36% | 7.09% | 15.9% |
|  | 5.25% | 32.3% | 20.8% |  | 2.13% | 8.56% | 16.2% |
|  | 5.39% | 34.2% | 17.4% |  | 2.22% | 7.96% | 14.9% |
| w/ DNT | 31.6% | 62.0% | 53.1% | w/ DNT | 6.44% | 17.8% | 42.0% |
|  | 33.0% | 60.9% | 51.8% |  | 6.15% | 16.8% | 41.3% |
|  | 33.6% | 60.4% | 47.9% |  | 6.11% | 16.2% | 38.4% |
