# Supplementary material for: Targeting chemotherapy-resistant leukemia by combining DNT cellular therapy with conventional chemotherapy
Source: J Exp Clin Cancer Res. 2018 Apr 24;37:88. doi: 10.1186/s13046-018-0756-9 (PMC5916833; doi:10.1186/s13046-018-0756-9)
Supplement: Supplementary file 5 — Table S2. Clinical characteristics of 13 AML patients whose AML blasts were used for in vitro assays. Abbreviations: WBC – White blood cell; BM – Bone marrow; FAB – The French-American-British classification of AML; MRC – Medical Research Council cytogenetic classification; MDS – Myelodysplastic syndrome; MK – Monosomal Karyotype; NPM1 – Nucleophosmin 1; FLT3-ITD – Fms related tyrosine kinase 3 - internal tandem duplication; FLT3-TKD – Fms related tyrosine kinase 3 - tyrosine kinase domain; BCR-ABL – Break point cluster region - Abelson murine leukemia viral oncogene homolog 1; RAR – Retinoic acid receptor. (DOCX 17 kb) [file 13046_2018_756_MOESM5_ESM.docx]

| **Sample**  **I.D.** | **Age** | **Sex** | **WBC Count** | **% Blast in BM** | **Cytogenetics** | **FAB** | **MRC** | **2^o^ to MDS** | **MK** | **NPM1** | **FLT3-ITD** | **FLT3-TKD** | **BCR-ABL** | **RAR** |
| --- | --- | --- | --- | --- | --- | --- | --- | --- | --- | --- | --- | --- | --- | --- |
| 150549 | 57 | F | 9.9 | 85 | 46,XX[20] | n.d. | intermediate (normal) | No | No | negative | negative | negative | n.d. | n.d. |
| 130696 | 73 | F | 311.4 | n.d. | 46,XX[20] | M4 | adverse | No | No | negative | intermediate | negative | n.d. | n.d. |
| 130926 | 67 | F | 40 | 92 | 46,XX[20] | M5a | intermediate (normal) | No | No | negative | negative | negative | n.d. | n.d. |
| 110162 | 26 | F | 59 | 89 | t(15;17) but otherwise unknown | M3 | favorable | No | n.d. | n.d. | n.d. | n.d. | n.d. | positive |
| 080043 | 27 | M | 246 | 90 | 47,XY,+4[20] | M0 | intermediate (abnormal) | No | No | n.d. | n.d. | n.d. | n.d. | n.d. |
| 150099 | 37 | M | 210.5 | 85 | 46,XY,inv(12)(q13q24.3),t(16;16)(p13.1;q21-23)[15] | M4Eo | intermediate (abnormal) | Yes | No | n.d. | n.d. | n.d. | n.d. | n.d. |
| 150273 | 70 | F | 16.3 | 58 | 46,XX del(9)(q13q22) | n.d. | n.d. | n.d. | n.d. | n.d. | n.d. | n.d. | n.d. | n.d. |
| 150935 | 21 | M | 92.3 | 64 | 46,XY,inv(16)(p13.1q22)[5]/46,XY[5] | M4Eo | n.d. | n.d. | n.d. | n.d. | n.d. | n.d. | n.d. | n.d. |
| 090240 | 54 | F | 2.5 | 60 | 52,XX,+2,+9,+10,+13,+14,+15 [20] | M1 | adverse | No | n.d. | n.d. | n.d. | n.d. | n.d. | n.d. |
| 120567 | 26 | M | 2 | n.d. | t(8;21) | n.d. | favorable | No | No | n.d. | n.d. | n.d. | n.d. | n.d. |
| 130607 | 75 | F | 227 | 80 | 46,XX[20] | M5b | intermediate (abnormal) | No | No | n.d. | n.d. | n.d. | n.d. | n.d. |
| 110080 | 71 | M | n.d. | n.d. | n.d. | n.d. | n.d. | No | n.d. | n.d. | n.d. | n.d. | n.d. | n.d. |
| 090271 | 65 | M | 1.6 | 90 | 46,XY | M4 | favorable | No | No | n.d. | n.d. | n.d. | n.d. | n.d. |
